# Cerebral hemorrhage due to tuberculosis meningitis: a rare case report and literature review

**DOI:** 10.18632/oncotarget.6528

**Published:** 2015-12-10

**Authors:** Hai Zou, Ke-Hua Pan, Hong-Ying Pan, Dong-Sheng Huang, Ming-Hua Zheng

**Affiliations:** ^1^ Department of Infection Diseases, Zhejiang Provincial People's Hospital, Hangzhou, China; ^2^ Department of Radiology, The First Affiliated Hospital of Wenzhou Medical University, Wenzhou, China; ^3^ Department of Hepatobiliary Surgery, Zhejiang Provincial People's Hospital, Hangzhou, China; ^4^ Department of Infection and Liver Diseases, Liver Research Center, The First Affiliated Hospital of Wenzhou Medical University, Wenzhou, China; ^5^ Institute of Hepatology, Wenzhou Medical University, Wenzhou, China

**Keywords:** intracerebral hemorrhage, leptomeningeal enhancement, tuberculosis meningitis, central nervous system

## Abstract

Tuberculosis (TB) is a common disease to threaten human health. TB of the central nervous system (CNS) is rare but the most serious type of systemic TB because of its high mortality rate, serious neurological complications and sequelae. In this case report, we describe a woman who presented with walking instability, intracerebral hemorrhage and leptomeningeal enhancement due to tuberculosis meningitis. The patient had no significant medical history and the initial clinical symptoms were walking instability. On analysis, the cerebrospinal fluid was colorless and transparent, the pressure was more than 400 mm H_2_O, there was lymphocytic pleocytosis, increased protein, and decreased glucose levels present. No tuberculosis or other bacteria were detected. The patient's brain computed tomography image showed intra-cerebral hemorrhage (ICH) and contrast magnetic resonance imaging showed ICH in the right frontal lob, and leptomeningeal enhancement. CNS TB is rare but has a high mortality rate. As this disease has no unique characteristics at first presentation such as epidemiology and obvious clinical manifestation, a diagnosis of CNS TB remains difficult.

## INTRODUCTION

Tuberculosis (TB) is a common disease to threaten human health. TB of the central nervous system (CNS; CNS TB) is rare but the most serious type of systemic TB because of its high mortality rate, serious neurological complications and sequelae. In this case report, we describe a women who presented walking instability, intracerebral hemorrhage and leptomeningeal enhancement due to tuberculosis meningitis.

## CASE PRESENTATION

**Figure 1:**
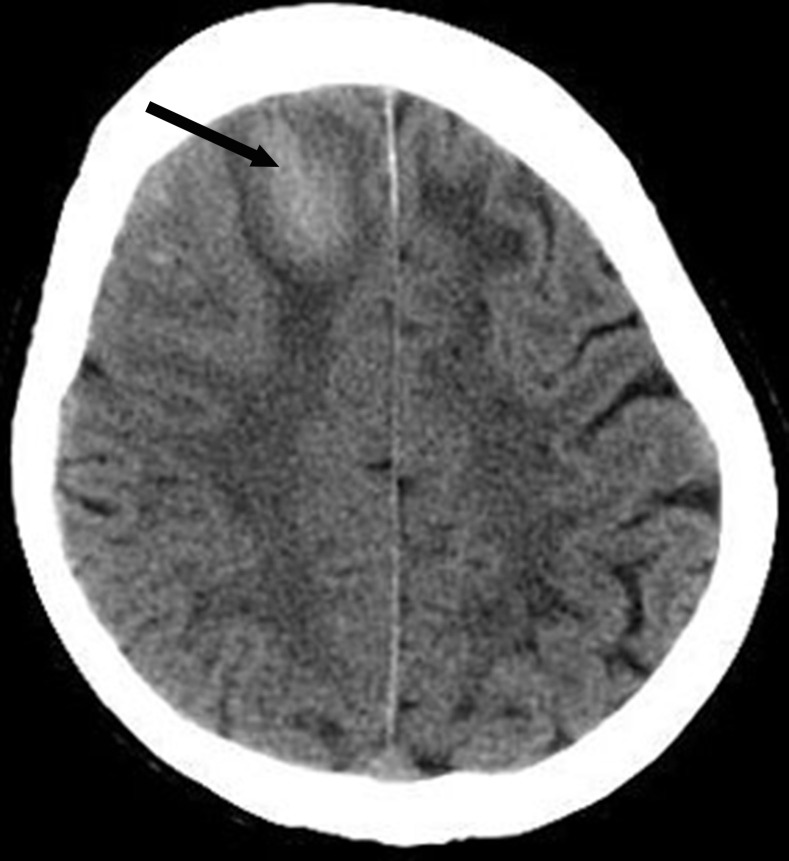
Noncontrast brain computed tomography shows a hematoma (arrow) in the right frontal lobe

**Figure 2:**
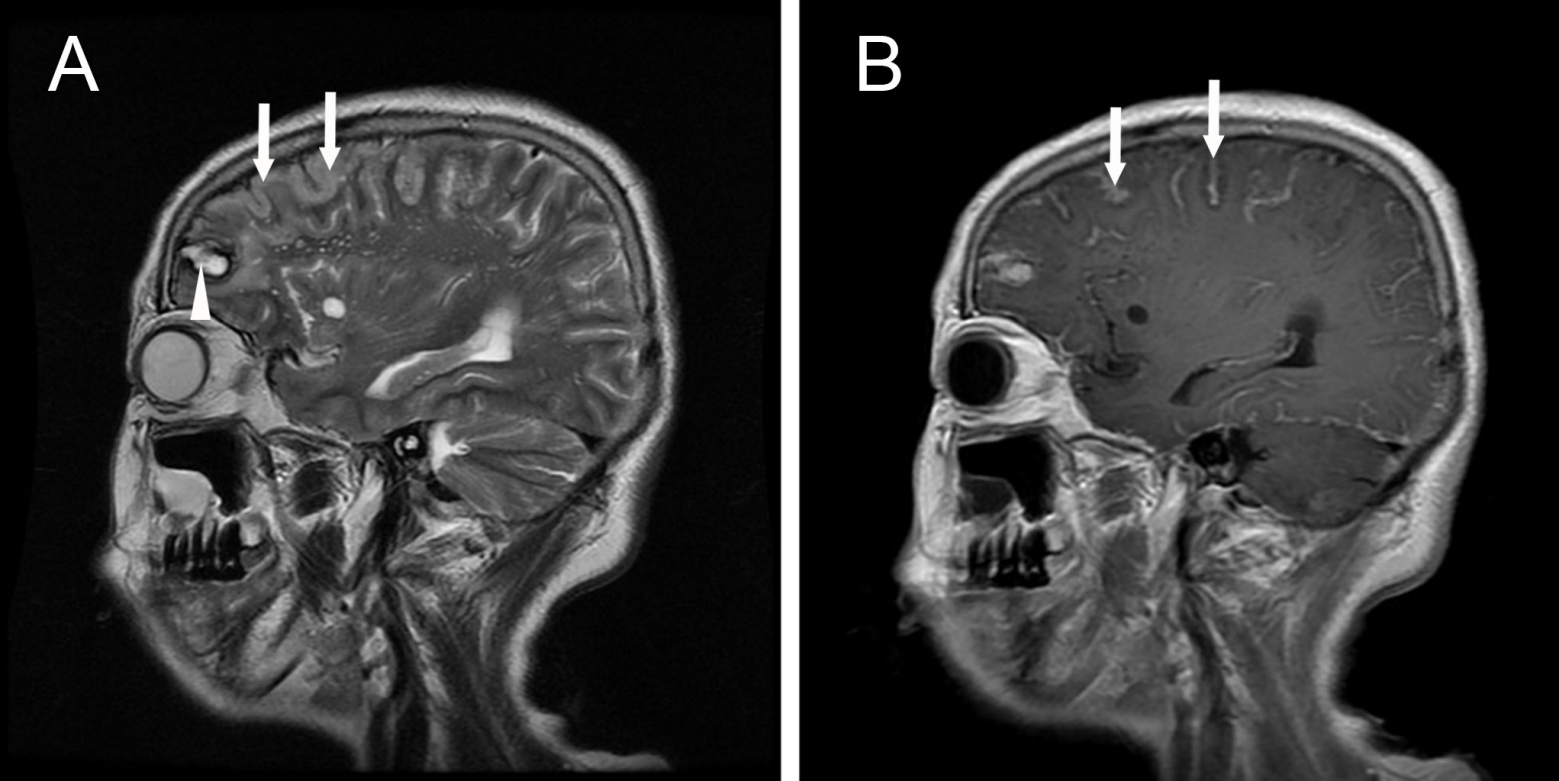
Sagittal brain magnetic resonance images **A.** T2-weighted magnetic resonance imaging shows a hematoma (arrow head) in the frontal lobe and cerebral gyrus swelling (arrows); **B.** T1-weighted magnetic resonance imaging with contrast shows abnormal leptomeningeal enhancement (arrows).

**Figure 3:**
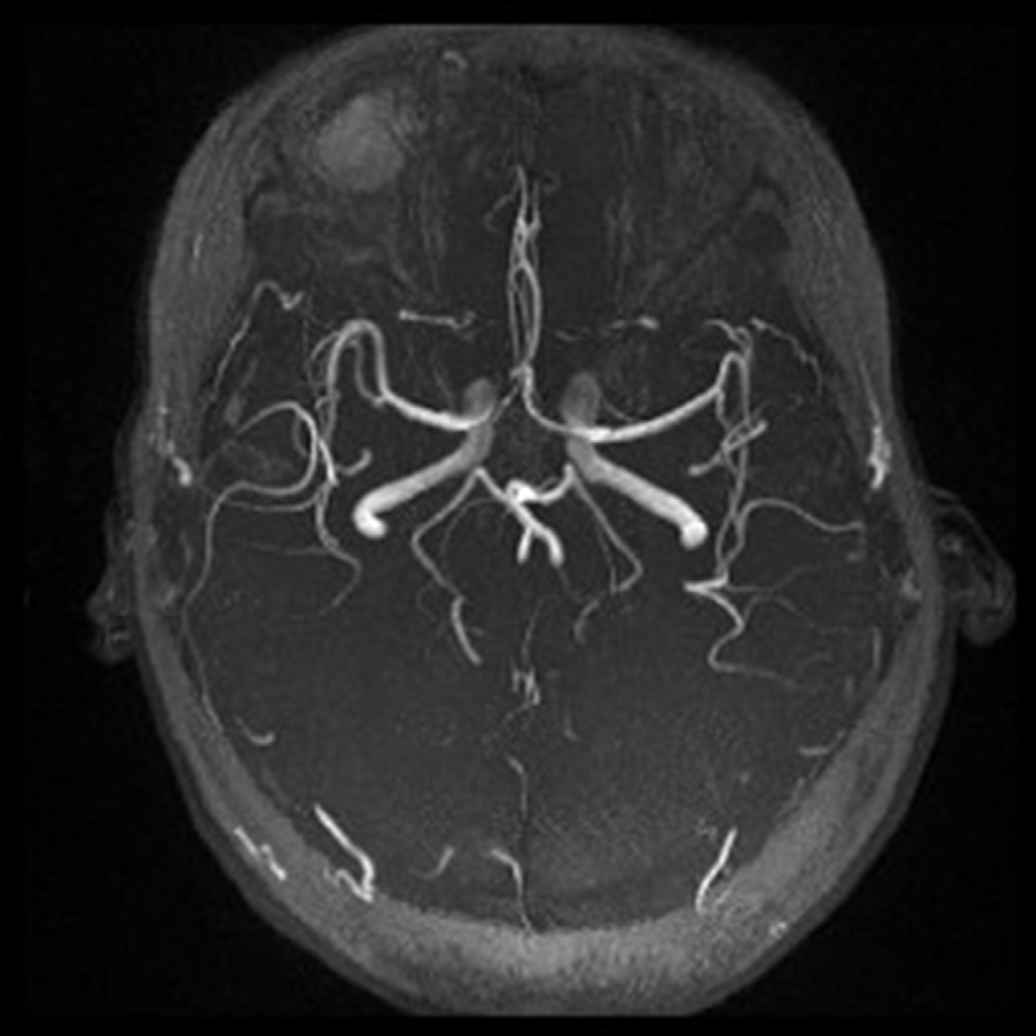
Magnetic resonance angiography showed no cerebral amyloid angiopathy, arteriovenous malformation and aneurysm

A 65-year-old woman presented with progressive walking instability, which first developed 7 months ago. This patient had no history of hypertension, diabetes, heart disease and tumor. She ignored the initial symptoms and did not go to hospital. Symptoms progressed and included headache, low-grade fever (temperature fluctuations from 100.4°F to 102.2°F), night sweats which persisted for 20 days with no nausea, vomiting, vertigo, malaise, visual changes, or weight loss. Physical examination showed that consciousness was clear, quarrel was not skewed, limb muscle strength and muscle tension was normal, Babinski's reflex was negative, meningeal stimulation was positive and other physical observations were unremarkable. Laboratory tests showed that her white blood cell count, coagulation function, human immunodeficiency virus and tumor marker tests were normal. Smears and cultures for ordinary bacteria and fungi were negative. Analysis of the CSF revealed colorless and transparent fluid, pressure was more than 400 mm H_2_O, white blood cell (WBC) count 60×10^6^/L, lymphocyte 97%, red blood cell (RBC) count 1240×10^6^/L, protein level 5.6g/L, glucose level 2.28mmol/L, chloride 125mmol/L, no tuberculosis and other bacteria were detected (Table 1). The patient's brain CT image showed a high-density shadow of right frontal lobe, CT value was 65 Hu, and indicated ICH (Figure 1). Contrast MRI of her brain showed ICH in the right frontal lob, and LME (Figures 2, 3). Chest X-ray was normal. As the CSF showed elevation of inflammatory biomarkers, meningeal irritation was positive, and MRI supported a suspected CNS infection, CNS infection was proposed as the most likely diagnosis in this previously healthy adult. After CSF was sent for culture, we selected ceftriaxone to implement our choice of anti-infective therapy. Isoniazid, pyrazinamide and rifampin were used as an anti-tuberculosis treatment and mannitol was used to reduce intracranial pressure. An acid-fast bacillus in CSF was not found and the CSF culture was assessed as negative. Unfortunately, 2 days after admission, the patient suddenly lost consciousness and showed corectasis. The patient accepted emergency craniotomy and 4 days after admission, the patient died. Histopathologic examination confirmed the diagnosis of tuberculous meningitis (TBM).

**Table 1:** Laboratory values of cerebrospinal fluid (CSF)

Variable of CSF	First time	Second time	Third time
Intracranial pressure (mmH_2_O)	200 (after mannitol intravenously guttae)	300	400
Color	Yellow	Yellow	Yellow
WBC (×10^6^/L)	16	9	60
LY (%)	96	96	97
RBC (×10^6^/L)	1960	2650	1240
Protein (g/L)	1.85	2.35	5.6
Glucose (mmol/L)	> 2.28	> 2.28	> 2.28
Chloride (mmol/L)	121.5	121	125

Note: First time: CSF of lumbar puncture performed on admission; Second time: CSF 3 days after admission; Third time: CSF 10 days after admission; WBC: White blood cell; LY: Lymphocyte; RBC: Red blood cell.

## DISCUSSION

TBM is the most common types of TB of the CNS. Hydrocephalus is the most frequent complication and is usually more prominent in children. Cerebral infarction is another common complication of basal meningitis which adds to the risk of morbidity and mortality [1]. Dastur et al. found infarcts in 41% of the specimens in an autopsy series of 100 patients [2]. Cerebral hemorrhage is, however, a rare complication. This patient was an old, previously healthy female with no known prior exposure to TB who presented with a complicated case of CNS TB leading to cerebral hemorrhage. CNS TB is encountered frequently in areas that continue to have high prevalence of tuberculosis and dissemination is very common in children and young adults. Previous literature reported that CNS TB occurs in all age groups, but 60-70% of patients are below the age of 20 years [3]. CNS TB is a unusual disease, which occurs in 5 to 10% of extrapulmonary TB cases, and accounts for approximately 1% of all all patients with TB [4]. With the onset of the human immunodeficiency virus pandemic, the incidence of CNS TB has increased [5–8], which accounts for approximately 10% of all patients with AIDS-related TB [9]. It is no longer a disease confined to underdeveloped and developing countries [5, 10].

CNS TB is the most serious type of systemic TB because of its high mortality rate, serious neurological complications and sequelae [11]. Clinical features of CNS TB commonly consist of a series of phases, beginning with low-grade fever, weak, sweating, and personality changes which may persist for a long time after which it may progress to a meningitic phase. The patient in this phase has more obvious neurologic features such as confusion, headache, even disturbance of consciousness. In the final phase, the patient may present with coma, seizures, and possibly stroke [12–14].

Our patient first presented with walking instability which had been present for a long time, then headache, low-grade fever and night sweats, and a CT examination showed ICH. Finally, she presented with coma. Generally, the clinical manifestations are non-specific, other brain diseases may also cause similar symptoms, including intracranial hemorrhage, bacterial meningitis, brain primary and metastatic tumor. Because of this, diagnosing CNS TB at an early stage may be very difficult owing to the unfamiliarity of doctors with this rare disease.

CSF examination is an important diagnostic method and the typical CSF shows elevated protein, low glucose,and elevated WBC with lymphocytic pleocytosis [15–16]. Elevated levels of adenosine deaminase in CSF and visualization of acid-fast bacilli in CSF smears may be early indicators, however, until recently a definitive diagnosis could only be established after growth and identification of mycobacterium tuberculosis in culture which could take 4-8 weeks and may provide a false negative result in 15-75% of cases [9, 17]. Currently, polymerase chain reaction (PCR) in CSF can diagnose TBM with a higher sensitivity than microscopic examination and cultures [18–20]. However, the possibility of false positive results is also a major concern when applying PCR tests for the diagnosis of TBM [21].

A CNS infection with mycobacterium tuberculosis can present either as a diffuse form (e.g., basal exudative leptomeningitis) or as a localized form (e.g., tuberculoma, abscess, or cerebritis). TBM often results in vasculitis and marked inflammatory reaction at the base of the brain, it is very likely that ICH in this case was secondary to the bleeding of the inflammatory vessels [22]. The CNS TB can mimic numerous other disease entities including nontuberculous bacterial, viral, parasitic meningitis, coccidioidomycosis, cryptococcosis), non-infectious inflammatory disease affecting the leptomeninges (e.g., rheumatoid disease, sarcoidosis), and meningeal carcinomatosis (e.g., breast carcinoma in adults) [23–25]. Imaging, therefore although a useful composite measure is nonspecific for the diagnosis of CNS TB. Contrast-enhanced MR imaging is generally considered to be superior to CT in the detection and assessment of CNS TB. Gadolinium-enhanced T1-weighted imaging demonstrates abnormal meningeal enhancement and is generally considered to be more sensitive than CT [23–25].

CNS TB remains a major cause of death or significant neurological disability. Consequently, prompt diagnosis and early treatment are of utmost importance to reduce morbidity and mortality. Clinical manifestation and laboratory tests are the cornerstones of early diagnosis, and MRI may be superior to CT in the detection and assessment of CNS TB.

## References

[1] Wasay M, Farooq S, Khowaja ZA, Bawa ZA, Ali SM, Awan S, Beg MA, Mehndiratta MM (2014). Cerebral infarction and tuberculoma in central nervous system tuberculosis: frequency and prognostic implications. J Neurol Neurosurg Psychiatry.

[2] Dastur DK, Lalitha VS, Udani PM, Parekh U (1970). The brain and meninges in tuberculous meningitis-gross pathology in 100 cases and pathogenesis. Neurol India.

[3] Kumar R, Jain R, Kaur A, Chhabra DK (2000). Brain stem tuberculosis in children. Br J Neurosurg.

[4] Cherian A, Thomas SV (2011). Central nervous system tuberculosis. Afr Health Sci.

[5] Saenz B, Hernandez-Pando R, Fragoso G, Bottasso O, Cardenas G (2013). The dual face of central nervous system tuberculosis: a new Janus Bifrons?. Tuberculosis (Edinb).

[6] Dai L, Mahajan SD, Guo C, Zhang T, Wang W, Li T, Jiang T, Wu H, Li N (2014). Spectrum of central nervous system disorders in hospitalized HIV/AIDS patients (2009-2011) at a major HIV/AIDS referral center in Beijing, China. J Neurol Sci.

[7] Chamie G, Marquez C, Luetkemeyer A (2014). HIV-associated central nervous system tuberculosis. Semin Neurol.

[8] Daniele B (2014). Characteristics of central nervous system tuberculosis in a low-incidence country: a series of 20 cases and a review of the literature. Jpn J Infect Dis.

[9] Garcia-Monco JC (1999). Central nervous system tuberculosis. Neurol Clin.

[10] Patkar D, Narang J, Yanamandala R, Lawande M, Shah GV (2012). Central nervous system tuberculosis: pathophysiology and imaging findings. Neuroimaging Clin N Am.

[11] Uysal G, Kose G, Guven A, Diren B (2001). Magnetic resonance imaging in diagnosis of childhood central nervous system tuberculosis. Infection.

[12] (2000). Targeted tuberculin testing and treatment of latent tuberculosis infection. This official statement of the American Thoracic Society was adopted by the ATS Board of Directors, July 1999. This is a Joint Statement of the American Thoracic Society (ATS) and the Centers for Disease Control and Prevention (CDC). This statement was endorsed by the Council of the Infectious Diseases Society of America. (IDSA), September 1999, and the sections of this statement. Am J Respir Crit Care Med.

[13] Chan KH, Cheung RT, Lee R, Mak W, Ho SL (2005). Cerebral infarcts complicating tuberculous meningitis. Cerebrovasc Dis.

[14] Farinha NJ, Razali KA, Holzel H, Morgan G, Novelli VM (2000). Tuberculosis of the central nervous system in children: a 20-year survey. J Infect.

[15] Solomons RS, Visser DH, Donald PR, Marais BJ, Schoeman JF, van Furth AM (2015). The diagnostic value of cerebrospinal fluid chemistry results in childhood tuberculous meningitis. Childs Nerv Syst.

[16] Philip N, William T, William DV (2015). Diagnosis of tuberculous meningitis: challenges and promises. Malays J Pathol.

[17] Villoria MF, Fortea F, Moreno S, Munoz L, Manero M, Benito C (1995). MR imaging and CT of central nervous system tuberculosis in the patient with AIDS. Radiol Clin North Am.

[18] Takahashi T, Tamura M, Takasu T, Kamei S (2013). [Current advancement of the PCR-based molecular diagnosis for tuberculous meningitis]. Rinsho Shinkeigaku.

[19] Takahashi T, Tamura M, Takasu T (2012). The PCR-Based Diagnosis of Central Nervous System Tuberculosis: Up to Date. Tuberc Res Treat.

[20] Kusum S, Aman S, Pallab R, Kumar SS, Manish M, Sudesh P, Subhash V, Meera S (2011). Multiplex PCR for rapid diagnosis of tuberculous meningitis. J Neurol.

[21] Marx GE, Chan ED (2011). Tuberculous meningitis: diagnosis and treatment overview. Tuberc Res Treat.

[22] Leonard JM, Des Prez RM (1990). Tuberculous meningitis. Infect Dis Clin North Am.

[23] Jinkins JR, Gupta R, Chang KH, Rodriguez-Carbajal J (1995). MR imaging of central nervous system tuberculosis. Radiol Clin North Am.

[24] Jamieson DH (1995). Imaging intracranial tuberculosis in childhood. Pediatr Radiol.

[25] Harisinghani MG, McLoud TC, Shepard JA, Ko JP, Shroff MM, Mueller PR (2000). Tuberculosis from head to toe. Radiographics.

