# Addendum: Cerebral hemorrhage due to tuberculosis meningitis: a rare case report and literature review

**DOI:** 10.18632/oncotarget.28284

**Published:** 2023-06-27

**Authors:** Hai Zou, Ke-Hua Pan, Hong-Ying Pan, Dong-Sheng Huang, Ming-Hua Zheng

**Affiliations:** ^1^Department of Infection Diseases, Zhejiang Provincial People’s Hospital, Hangzhou, China; ^2^Department of Radiology, The First Affiliated Hospital of Wenzhou Medical University, Wenzhou, China; ^3^Department of Hepatobiliary Surgery, Zhejiang Provincial People’s Hospital, Hangzhou, China; ^4^Department of Infection and Liver Diseases, Liver Research Center, The First Affiliated Hospital of Wenzhou Medical University, Wenzhou, China; ^5^Institute of Hepatology, Wenzhou Medical University, Wenzhou, China; ^*^Co-first author

**This article has an addendum:** Informed patient consent was obtained from all patients.

Original article: Oncotarget. 2015; 6:45005–45009. 45005-45009. https://doi.org/10.18632/oncotarget.6528

